# Restrictive vs. Liberal Red Blood Cell Transfusion Strategy in Patients With Acute Myocardial Infarction and Anemia: A Systematic Review and Meta-Analysis

**DOI:** 10.3389/fcvm.2021.736163

**Published:** 2021-11-16

**Authors:** Yeshen Zhang, Zhengrong Xu, Yuming Huang, Qirao Ye, Nianjin Xie, Lihuan Zeng, Xingji Lian, Yining Dai, Jiyan Chen, Pengcheng He, Ning Tan, Yuanhui Liu

**Affiliations:** ^1^Department of Cardiology, Guangdong Cardiovascular Institute, Guangdong Provincial Key Laboratory of Coronary Heart Disease Prevention, Guangdong Provincial People's Hospital, Guangdong Academy of Medical Sciences, Guangzhou, China; ^2^Department of Cardiology, People's Hospital of Baoan Shenzhen, Shenzhen, China; ^3^Department of Catheterization Lab, Guangdong Cardiovascular Institute, Guangdong Provincial Key Laboratory of South China Structural Heart Disease, Guangdong Provincial People's Hospital, Guangdong Academy of Medical Sciences, Guangzhou, China; ^4^Department of Cardiology, Shangyou People's Hospital, Ganzhou, China; ^5^Department of Nephrology, The First Affiliated Hospital, Sun Yat-sen University, Key Laboratory of Nephrology, National Health Commission of China and Guangdong Province, Guangzhou, China

**Keywords:** acute myocardial infarction, anemia, restrictive blood transfusion, liberal blood transfusion, meta-analysis

## Abstract

**Objective:** Anemia is frequent in patients with acute myocardial infarction (AMI), and the optimal red blood cell transfusion strategy for AMI patients with anemia is still controversial. We aimed to compare the efficacy of restrictive and liberal red cell transfusion strategies in AMI patients with anemia.

**Methods:** We systematically searched PubMed, EMBASE, Web of Science, Cochrane Library, and Clinicaltrials.gov, from their inception until March 2021. Studies designed to compare the efficacy between restrictive and liberal red blood cell transfusion strategies in patients with AMI were included. The primary outcome was all-cause mortality, including overall mortality, in-hospital or follow-up mortality. Risk ratios (RR) with 95% confidence intervals (CI) were presented and pooled by random-effects models.

**Results:** The search yielded a total of 6,630 participants in six studies. A total of 2,008 patients received restrictive red blood cell transfusion while 4,622 patients were given liberal red blood cell transfusion. No difference was found in overall mortality and follow-up mortality between restrictive and liberal transfusion groups (RR = 1.07, 95% CI = 0.82–1.40, *P* = 0.62; RR = 0.89, 95% CI = 0.56–1.42, *P* = 0.62). However, restrictive transfusion tended to have a higher risk of in-hospital mortality compared with liberal transfusion (RR = 1.22, 95% CI = 1.00–1.50, *P* = 0.05). No secondary outcomes, including follow-up reinfarction, stroke, and acute heart failure, differed significantly between the two groups. In addition, subgroup analysis showed no differences in overall mortality between the two groups based on sample size and design.

**Conclusion:** Restrictive and liberal red blood cell transfusion have a similar effect on overall mortality and follow-up mortality in AMI patients with anemia. However, restrictive transfusion tended to have a higher risk of in-hospital mortality compared with liberal transfusion. The findings suggest that transfusion strategy should be further evaluated in future studies.

## Introduction

Anemia is frequent in patients with acute myocardial infarction (AMI), with the reported rates of 15–43% (1), because of invasive procedures and antithrombotic therapy (2). Anemia increases the incidence of adverse cardiovascular events, including short- and long-term mortality among patients with AMI (1, 3). Red blood cell (RBC) transfusion increases oxygen delivery, rapidly improves symptoms in patients with acute myocardial ischemia, and is commonly used in clinical practice (4). However, inappropriate blood transfusion may lead to circulatory overload and increased thrombogenicity, which can worsen the clinical outcomes (5–7). Therefore, it is essential to select the optimal transfusion strategy in AMI patients with anemia.

To date, the risks and benefits of optimal transfusion strategy, liberal or restrictive transfusion, remain unclear in such patients. Although several meta-analyses about the transfusion strategies have been published, they did not examine the subgroup of AMI patients with anemia (8–10). Observational studies have yielded conflicting results (11–13) and only two previous small randomized clinical trials (RCTs) (including 45 and 110 patients) have compared transfusion strategies in patients with AMI (14, 15). Recently, the first multicenter RCT with a relatively large sample size has compared liberal and restrictive RBC transfusion strategies in such settings (16), and the results showed that the restrictive transfusion resulted in a non-inferior rate of adverse outcomes after 30 days.

However, no meta-analysis has specifically compared the outcomes of different transfusion strategies in AMI patients with anemia. Therefore, we performed the present meta-analysis to assess the efficacy of restrictive transfusion vs. liberal transfusion in such patients.

## Methods

This meta-analysis was performed in accordance with the PRISMA guidelines (17). The protocol was registered with PROSPERO in September 2020, number CRD420202 04670. Ethical approval and patient consent were not required because this study was based on previous studies.

### Literature Search Strategy

We systematically searched five electronic databases, including PubMed, EMBASE, Web of Science, Cochrane Library, and Clinicaltrials.gov, from their inception until March 2021 for studies designed to compare the efficacy between restrictive and liberal transfusion in AMI patients with anemia. Restrictive transfusion was defined as hemoglobin threshold ≤ 8 g/dL or hematocrit ≤ 24%, while liberal transfusion was defined as hemoglobin threshold ≤ 10 g/dL or hematocrit ≤ 30% (18). In order to systematically search these electronic databases, search terms were constructed as follows: (Transfusion OR Blood transfusion OR Red blood cell transfusion) AND (Myocardial infarction OR Acute myocardial infarction OR ST-segment elevation myocardial infarction OR Non-ST-segment elevation myocardial infarction OR Acute coronary syndrome OR Percutaneous coronary intervention). The search was not restricted for trials by type, language, or publication status. To screen for additional studies, the reference lists of the included articles and previous relevant meta-analyses were also carefully scanned. Additionally, the major international cardiology meetings (the European Society of Cardiology, the American Heart Association, and the American College of Cardiology) were also searched for relevant conference abstracts with complete results.

### Study Selection

The study had to satisfy the following criteria to be included: (1) AMI patients suffering from anemia including pre-existing anemia and hospital-acquired anemia; (2) one group received the liberal and the other group received the restrictive red blood cell transfusion strategy; and (3) data regarding the risk of in-hospital or follow-up mortality and follow-up reinfarction, stroke, or acute heart failure.

Studies designed to compare the efficacy between blood transfusion and non-transfusion patients with AMI but without separate data on different transfusion strategies were excluded. In addition, other exclusion criteria were as follows: (1) reviews, meta-analyses, letters, and conferences; (2) *in vitro* or preclinical animal studies; (3) enrolled pediatric patients; and (4) duplicate data. Three authors (YSZ, ZRX, and QRY) independently screened the articles based on titles and abstracts. We solved the disagreements and reached a consensus through discussion or arbitration by the fourth reviewer (YMH).

### Outcomes

The primary endpoint was all-cause mortality, including overall mortality, in-hospital mortality, or follow-up mortality (up to 6 months). The secondary outcomes were follow-up reinfarction, stroke, and acute heart failure.

### Data Extraction and Quality Assessment

Data extraction was independently carried out by three reviewers (YSZ, ZRX, and QRY) on the basis of prespecified extraction criteria. The following information was extracted from each included study: author, publication date, country, study design, sample size, patients' characteristics, transfusion strategy, and outcomes. The fourth reviewer (YMH) checked for discrepancies and helped to settle the disagreements.

The quality of included studies was also independently evaluated by three reviewers (YSZ, ZRX, and QRY). The Cochrane Collaboration's risk of bias tool was used to assess the quality of RCTs (19), which comprised assessment of selection bias and other bias. To evaluate the quality of cohort studies, we used the Newcastle-Ottawa Scale (20), which consists of eight items about sampling methods, comparability, and accuracy of results. A study with a NOS score fewer than 4 was considered to be of poor quality, while the NOS score of a study equal or more than 7 was considered to be of good quality (21, 22). The disagreements were resolved through discussion among the reviewers and judged by the fourth reviewer (YMH).

### Statistical Analysis

The statistical analyses were carried out using Review Manager (version 5.3) and R software (version 4.0.2). Heterogeneity of studies was assessed using Q statistics and *I*^2^. *I*^2^ value <25% indicated low heterogeneity, 25–50% denoted moderate heterogeneity, while the values over 50% defined severe heterogeneity. In order to minimize bias due to methodological differences between the studies, random-effects models were selected (23, 24). Differences in results of all of the included studies were represented in the form of forest plots. Overall mortality was analyzed by subgroup analysis based on the study design and the number of cases. The sensitivity analysis of omission was carried out to evaluate the robustness of the results. Additionally, the funnel plot analysis and Egger's test were used to evaluate the publication bias. Risk ratios (RR) with 95% confidence intervals (CI) were presented and pooled by random-effects models. All the differences in the two-tailed test with *P* < 0.05 were considered to be statistically significant.

## Results

### Search Results

**Figure 1:**
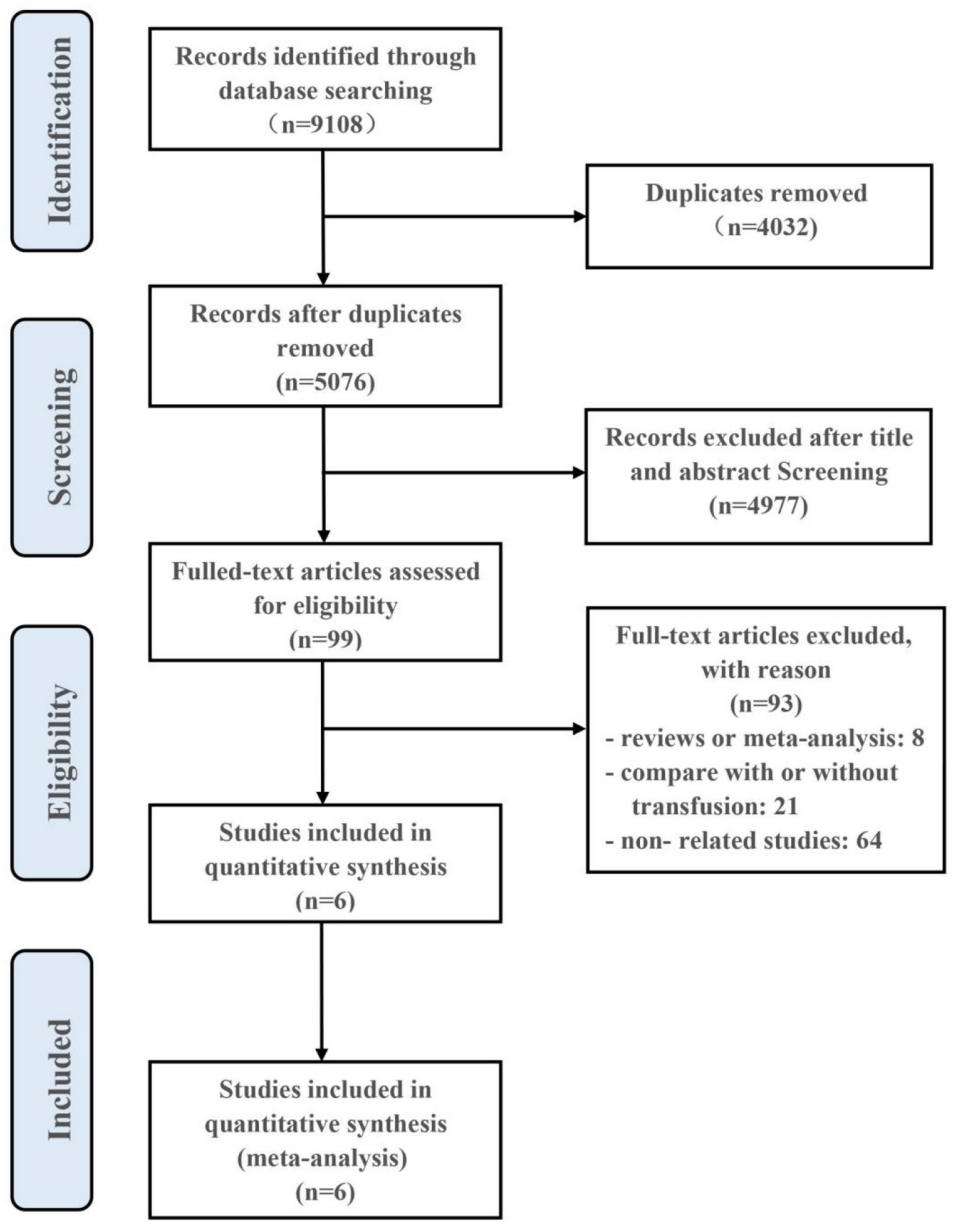
The flow diagram indicated the process of study selection.

Initially, 9,108 articles were found through systematic search. After removing duplicates, 4,977 articles were excluded based on title and abstract screening. Then, the full texts of 99 studies were reviewed to evaluate whether they met the inclusion criteria. Consequently, six studies meeting all of the criteria were finally included. The flow chart of the research selection process is shown in Figure 1.

### Study Characteristics and Quality Assessment

A total of six articles (6,630 patients in total), published from 2001 to 2021, were included (4, 13–16, 25). Three of the included studies were RCTs and three were cohort studies (one prospective study, two retrospective studies). Five articles included patients who received blood transfusions in the AMI setting, and only one article included patients with blood transfusions in the setting of acute coronary syndrome or stable angina (but data about AMI could be extracted). Among the included patients, the average age ranged from 69.0 to 79.5 years, and 2,949 (44.5%) were men (Table 1). In addition, 2008 AMI patients with anemia received restrictive red blood cell transfusion, while 4,622 patients were given liberal red blood cell transfusion.

**Table 1:** Characteristics of included studies in this meta-analysis.

**Study**	**Region**	**Study design**	**Transfused patients**	**Types of MI**	**Restrictive strategy (Transfusion threshold)**	**Liberal strategy(Transfusion threshold)**	**Average age (years)**		**Male No. (%)**		**Baseline Hb (g/dl)/Hct (%)**		**Follow-up (months)**	**Primary outcome**
							**RT**	**LT**	**RT**	**LT**	**RT**	**LT**		
Wu et al. (13)	USA	Cohort	1,356	STEMI Non-STEMI	Hct ≤ 24%	Hct ≤ 30%	79.5	79.4	112 (41)	398 (37)	–	–	1	30-day Mortality
Alexander et al. (4)	USA	Cohort	4,291	Non-STEMI	Hct ≤ 24%	Hct ≤ 30%	76.0	76.6	578 (45)	1,303 (44)	–/29.0	–/33.8	In hospital	In-hospital Mortality
Aronson et al. (25)	USA	Cohort	192	STEMI Non-STEMI	Hb ≤ 8 g/dl	Hb > 8 g/dl	69.0	69.0	21 (58)	89 (57)	11.8/-	11.8/-	6	All-cause mortality Composite: Mortality /MI/HF
Cooper et al. (15)	USA	RCT	45	STEMI Non-STEMI	Hct <24%	Hct <30%	70.3	76.4	13 (54)	10 (48)	–/27.5	–/26.9	1	Composite: In-hospital death, recurrent MI, new or worsening congestive heart failure
Carson et al. (14)	USA	RCT	80	STEMI Non-STEMI	Hb <8 g/dl	Hb <10 g/dl	74.3	67.3	19 (49)	21 (51)	8.97/–	9.18/–	1	Composite: all-cause mortality, MI, or, unscheduled coronary revascularisation
Ducrocq et al. (16)	France Spain	RCT	666	STEMI Non-STEMI	Hb ≤ 8 g/dl	Hb ≤ 10 g/dl	78.0	76.0	201 (59)	184 (57)	9.0/–	9.1/–	1	Major adverse cardiovascular events

*MI, Myocardial infarction; STEMI, ST-segment elevation myocardial infarction; Non-STEMI, Non-ST-segment elevation myocardial infarction; Hct, Hematocrit; Hb, Hemoglobin; RT, Restrictive transfusion; LT, Liberal transfusion; HF, Heart failure*.

All of the included studies were of high quality, as confirmed by the Newcastle-Ottawa Scale and the Cochrane Collaboration's risk of bias tool. The quality assessment of the eligible studies is shown in Table 2, Supplementary Figure 1.

**Table 2:** Results of quality assessment using the Newcastle-Ottawa Scale for included studies (Cohort Studies).

**Study**	**Selection**				**Comparability**	**Exposure**			**Scores**
	**Adequate definition of cases**	**Representativeness of the cases**	**Selection of controls**	**Definition of controls**	**Control for important factor^a^**	**Ascertainment of exposure**	**Same method of ascertainment for cases and controls**	**Non-response rate**	
Wu et al. (13)	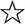	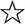	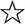	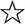	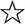 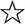	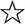	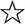	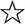	9
Alexander et al. (4)	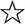	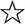	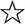	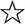	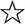 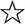	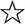	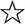	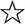	9
Aronson et al. (25)	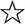	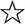	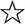	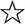	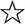 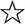	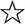	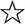	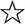	9

a *a maximum of 2 stars can be allotted in this category, one for age, the other for other controlled factors*.

### Association of the Transfusion Strategy and Outcome

**Figure 2:**
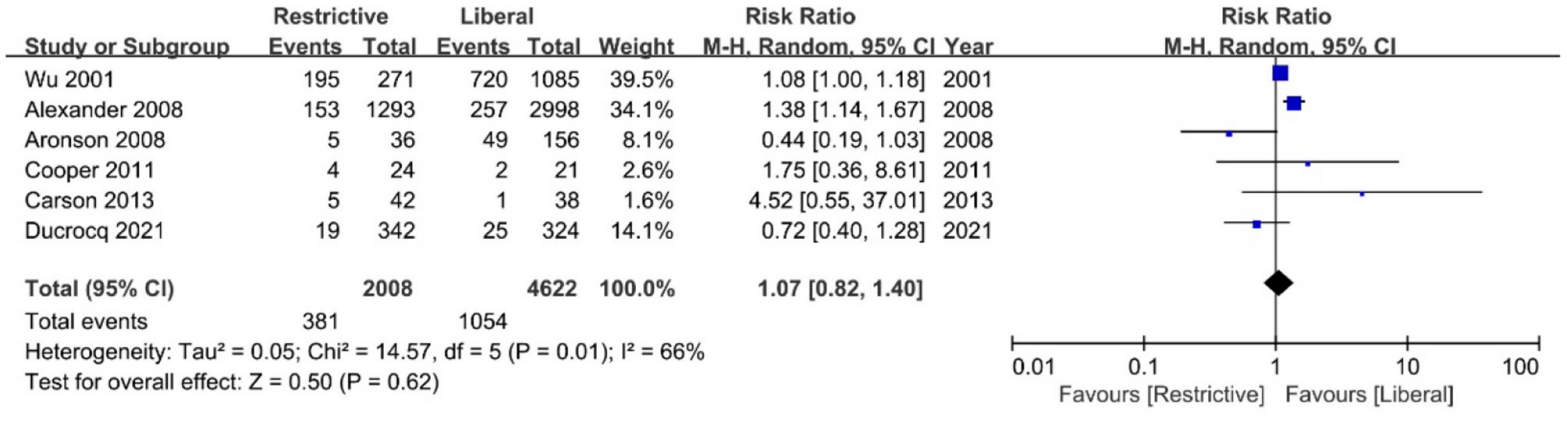
Forest plot for the association between blood transfusion strategies with overall mortality.

There was no statistically significant difference in overall mortality between the restrictive transfusion group and the liberal transfusion group (RR, 1.07 [95% CI = 0.82–1.40]; *P* = 0.62; *I*^2^ = 66%), with the severe heterogeneity observed (Figure 2). Three studies and four studies evaluated the relationship between blood transfusion strategies and in-hospital mortality or follow-up mortality, respectively. There was also no significant difference in follow-up mortality between the two groups (RR, 0.89 [95% CI = 0.56–1.42]; *P* = 0.62; *I*^2^ = 50%), while restrictive transfusion tended to have a higher risk of in-hospital mortality compared with liberal transfusion (RR, 1.22 [95% CI = 1.00–1.50]; *P* = 0.05; *I*^2^ = 41%) (Supplementary Figure 2).

**Figure 3:**
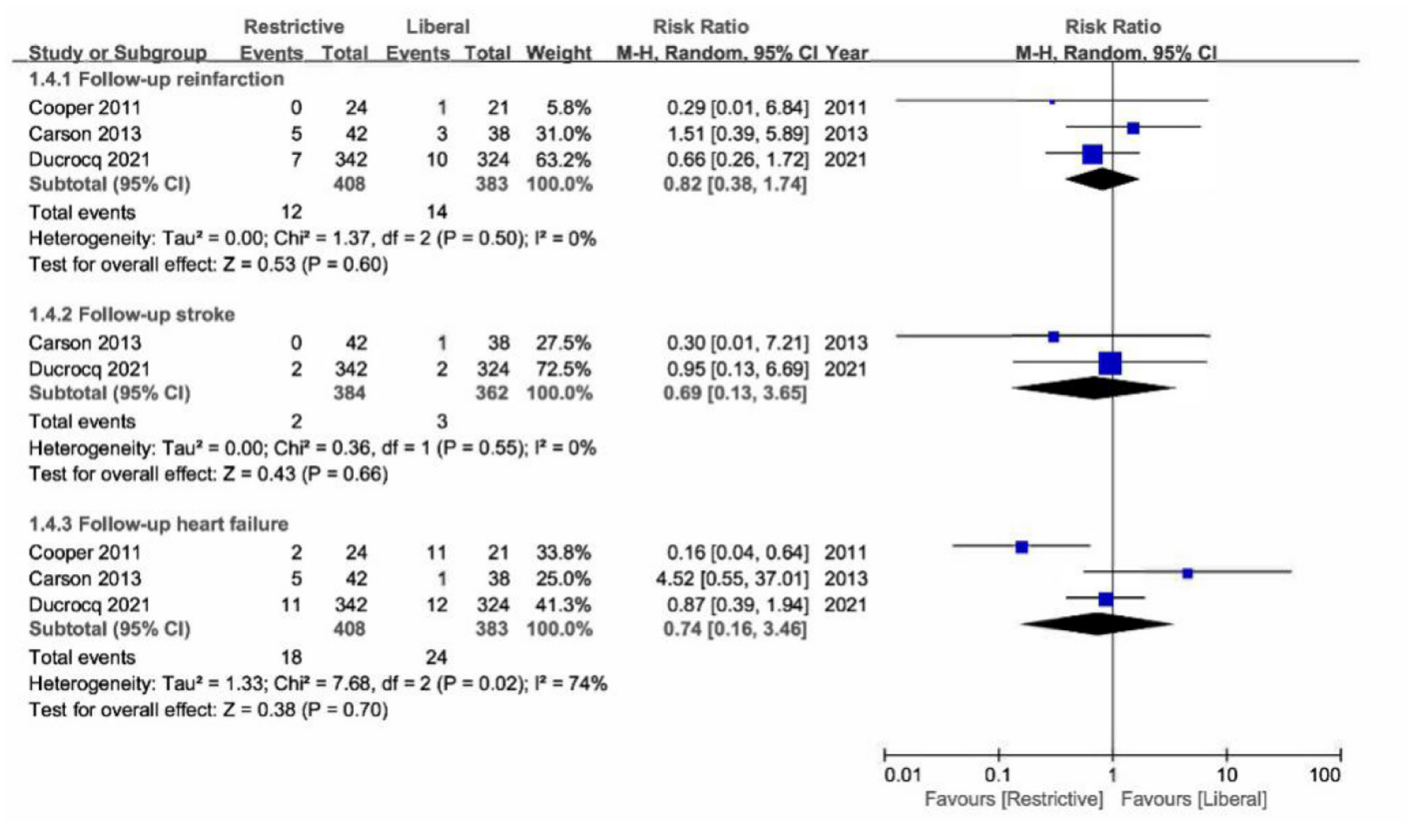
Forest plot for the association between blood transfusion strategies with secondary outcomes.

In addition, no obvious difference was found in the secondary outcomes of follow-up reinfarction (RR, 0.82 [95% CI = 0.38–1.74]; *P* = 0.60; *I*^2^ = 0%), stroke (RR, 0.69 [95% CI = 0.13–3.65]; *P* = 0.66; *I*^2^ = 0%), and acute heart failure (RR, 0.74 [95% CI = 0.16–3.46]; *P* = 0.70; *I*^2^ = 74%) between the restrictive transfusion group and the liberal transfusion group (Figure 3).

### Subgroup Analysis

**Figure 4:**
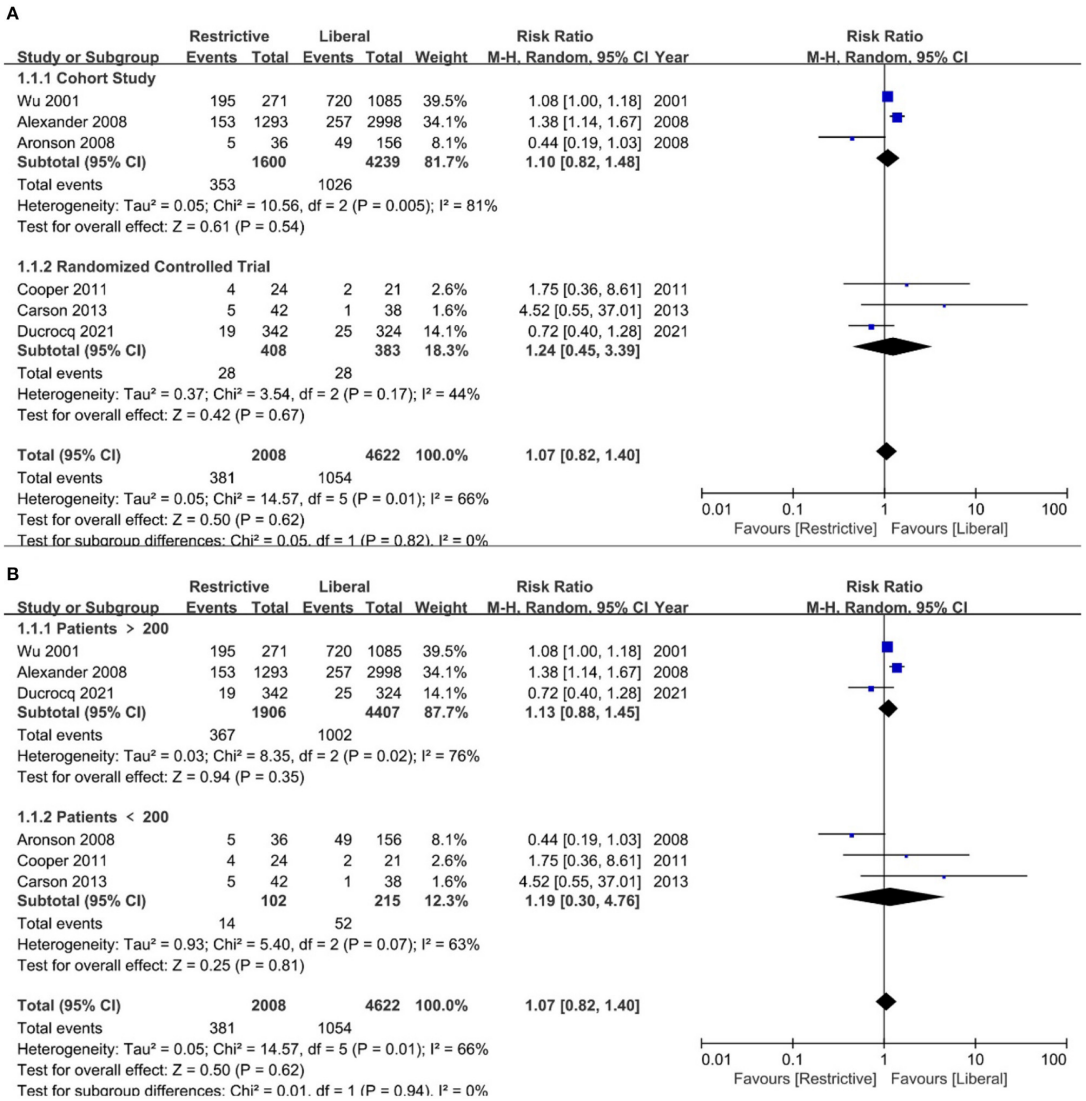
Subgroup analysis of overall mortality for blood transfusion strategies according to study design and sample size. **(A)**: Study design; **(B)**: study sample size.

A predefined subgroup analysis of overall mortality according to study design and sample size was performed. The results remained similar to the primary analysis (Figures 4A,B).

### Publication Bias and Sensitivity Analysis

The asymmetric distribution of funnel plot suggested that there could be publication bias among the included studies (Supplementary Figure 3). However, no publication bias was detected through Egger's test, with *P*-value being 0.7829 for overall mortality. The sensitivity analysis of omission showed that removing a single study each time had no significant effect on the results (Supplementary Figure 4).

## Discussion

The present study showed no differences in overall mortality and follow-up mortality between the restrictive and liberal transfusion in AMI patients with anemia. Moreover, no secondary outcomes differed significantly between the two groups. However, restrictive transfusion tended to have a higher risk of in-hospital mortality compared with liberal transfusion.

Several studies have shown that blood transfusion was related to the increased risk of repeated myocardial infarction and short- and long-term mortality, especially for patients suffering from AMI (26–28). A meta-analysis including 10 studies concluded that blood transfusion increased incidence of all-cause death in patients suffering from MI compared with the absence of blood transfusion (12). Conversely, the findings of a multicenter study have shown that blood transfusion was associated with lower risk of in-hospital mortality in propensity-matched patients with AMI and indicated that previous observational reports of increased mortality with transfusion may have been influenced by selection bias (29). The 2020 ESC Guideline for the management of patients with Non-ST-Elevation acute coronary syndromes recommended that these patients with anemia should not routinely receive RBC transfusion when hematocrit is above 25% or hemoglobin levels above 8 g/dL (class IIb, level of evidence C) (30). Given the results above, we should be cautious about the blood transfusion strategy for AMI patients with anemia in clinical practice.

RBC transfusion can rapidly increase oxygen delivery and improve some symptoms of the AMI patients with anemia (4). However, the threshold for blood transfusion is still controversial in this population. Previous meta-analysis which only included few patients with AMI (*n* = 154) found that there were no differences in mortality between restrictive and liberal transfusion strategies in subgroup of patients with AMI, but restrictive transfusion strategy was not recommended because of insufficient evidence (9). Another meta-analysis of 11 RCTs also showed that it may not be safe to use a restrictive transfusion threshold of <80 g/L in patients with ongoing acute coronary syndrome or chronic cardiovascular disease because restrictive blood transfusion can increase the risk of acute coronary syndrome; however, there was no difference in 30-day mortality between the two transfusion strategies (8). There had some deficiencies for previous meta-analysis and researches. Firstly, a limited number of studies with small sample sizes, comparing restrictive transfusion with liberal transfusion in patients with AMI were included, which tended to overestimate intervention effect estimates and could not make any relevant conclusions regarding intervention effects. Secondly, some of the abovementioned studies were not focus on the patients with AMI, which were different from other patient populations because of acute myocardial ischemia. These analyses included not only patients with myocardial infarction but also patients with other types of coronary artery disease. Until now, there has been insufficient evidence to prove which transfusion strategy is the optimal in patients with AMI. Therefore, we conducted a meta-analysis focus on the patients with AMI to compare the efficacy of restrictive and liberal transfusion strategies.

To date, three RCTs have evaluated the restrictive and liberal transfusion strategies in AMI patients with anemia. Of those, two randomized pilot trials were performed to compare the efficacy between restrictive and liberal transfusion, but their findings were inconsistent. In one pilot trial, the results showed that the liberal transfusion strategy may be associated with worse clinical outcomes (15). In contrast, another pilot trial demonstrated that the liberal transfusion strategy, compared with a more restrictive strategy, was able to reduce the incidence of major cardiac events and deaths among anemic patients with acute coronary syndrome or stable angina (14). Recently, the first multicenter RCT compared liberal vs. restrictive RBC transfusion strategies in patients with AMI and anemia (16), showing that the restrictive transfusion strategy not only was no less clinically effective than the liberal transfusion strategy, but it also saved blood. This RCT provided a more sufficient basis for clinical practice.

Based on the new researches in our meta-analysis, a half of the included studies were RCTs, which reduced the impact from confounding and other inherent bias that could affect the outcomes. Moreover, we only included studies with AMI patients, which led to robust pooled results to estimate the benefit of restrictive or liberal RBC transfusion in AMI patients. Our meta-analysis found that restrictive RBC transfusion for AMI patients did not affect overall mortality and the incidence of follow-up reinfarction, stroke, and acute heart failure compared with liberal RBC transfusion, although liberal RBC transfusion slightly reduced in-hospital mortality. The possible reason for this observation is that cardiac ischemia can occur at lower hemoglobin levels in patients with coronary heart disease (31), and liberal blood transfusion can alleviate myocardial ischemia, thereby reducing the risk of death. In addition, most of the patients included in present meta-analysis were elderly patients with limited compensatory capacity of the heart and lungs when they suffered from myocardial infarction, so restrictive blood transfusion may increase in-hospital mortality.

Although liberal RBC transfusion may slightly reduce in-hospital mortality, there are many potential risks associated with more liberal transfusion strategy. First, liberal transfusion may increase the risk of hospital-acquired infections, compared with restrictive transfusion (32). Second, liberal transfusion is associated with circulatory overload, which could increase the burden on the heart and energy expenditure. Third, the increase of RBC transfusion leads to an increase in platelet reactivity, which may be related to increased incidence of myocardial reinfarction and other advert myocardial events (27, 33). Therefore, transfusion strategy for AMI and anemic patients should be evaluated in the more future researches.

### Limitations

The present meta-analysis still has several limitations. Firstly, although half of the included studies were RCTs, we also included three observational studies. The inherent bias of those studies could have affected the pooled outcomes. Secondly, some data about the outcomes (such as advert myocardial events) were not completely available. So future studies should focus more on the comparison of follow-up advert myocardial events between restrictive and liberal transfusion in AMI patients with anemia. Thirdly, due to the lack of data on transfusion adverse reactions in the included studies, we were unable to evaluate the effects of different transfusion strategies on transfusion adverse reactions in AMI patients with anemia. Fourthly, we performed a subgroup analysis of overall mortality for blood transfusion strategies according to study design. However, our study did not present the results of other endpoints according to study design because available data is insufficient. Fifthly, the heterogeneity between trials was high. The most important reason may be the difference in patient populations, because anemic patients include those with pre-existing anemia and hospital-acquired anemia caused by various reasons. Differences in methods between RCTs and cohort studies, integration of RCTs and cohort studies may also lead to greater heterogeneity. In addition, the variation in case number, follow-up duration, setting of outcome indicators also leads to heterogeneity. Finally, most of the patients included were American and older. Therefore, caution should be exercised when extending the conclusion of this study to a wider population. Although there were some limitations in this meta-analysis, the findings of this study could help to better understand the effects of different RBC transfusion strategies on patients suffering from AMI and anemia. More RCTs should be conducted in the future to verify the results of this meta-analysis.

## Conclusion

Restrictive and liberal red blood cell transfusion have a similar effect on overall mortality and follow-up mortality in AMI patients with anemia. However, restrictive transfusion tended to have a higher risk of in-hospital mortality compared with liberal transfusion. The findings suggest that transfusion strategy should be further evaluated in future studies.

## Data Availability Statement

The original contributions presented in the study are included in the article/Supplementary Material, further inquiries can be directed to the corresponding author/s.

## Author Contributions

YZ, ZX, YH, and QY: contributed equally to this work. NT and YL: conceived the study and designed the protocol. YZ and ZX: performed the literature search. ZX, YZ, YH, and QY: selected the studies and extracted the relevant information. YZ, ZX, and QY: synthesized the data. YL, YZ, and ZX: wrote the first draft of the paper. All authors critically revised successive drafts of the paper and approved the final version.

## Funding

This work was supported by the Shuangqing Talent Program Project of Guangdong Provincial people's Hospital [Grant Nos. KJ012019095 to YL and KJ012019084 to PH], China Youth Research Funding (2017-CCA-VG-02), and Guangdong Provincial People's Hospital Clinical Research Fund (Y012018085).

## Conflict of Interest

The authors declare that the research was conducted in the absence of any commercial or financial relationships that could be construed as a potential conflict of interest.

## Publisher's Note

All claims expressed in this article are solely those of the authors and do not necessarily represent those of their affiliated organizations, or those of the publisher, the editors and the reviewers. Any product that may be evaluated in this article, or claim that may be made by its manufacturer, is not guaranteed or endorsed by the publisher.
