# Supplementary material for: Restrictive vs. Liberal Red Blood Cell Transfusion Strategy in Patients With Acute Myocardial Infarction and Anemia: A Systematic Review and Meta-Analysis
Source: Front Cardiovasc Med. 2021 Nov 16;8:736163. doi: 10.3389/fcvm.2021.736163 (PMC8636896; doi:10.3389/fcvm.2021.736163)

**/SUPPLEMENTAL MATERIAL**

[Search strategy 1](#_Toc4986)

[Definition 2](#_Toc17800)

[Figure S1 Results of quality assessment using the Cochrane Collaboration’s risk of bias tool (Randomized Controlled Trial) 4](#_Toc20503)

[Figure S2 Forest plot for the association between blood transfusion strategies with in-hospital and follow-up mortality 5](#_Toc29235)

[Figure S3 Funnel plot analysis of potential publication bias 6](#_Toc6840)

[Figure S4 A leave-one-out sensitivity analysis of this meta-analysis 7](#_Toc14347)

# Search strategy

- 1. **Search strategy for PubMed:**

(((Transfusion[Title/Abstract]) OR (Blood transfusion[Title/Abstract])) OR (Red blood cell transfusion[Title/Abstract]))) AND ((((((Myocardial infarction[Title/Abstract]) OR (Acute myocardial infarction[Title/Abstract])) OR (ST-segment elevation myocardial infarction[Title/Abstract])) OR (Non-ST-segment elevation myocardial infarction[Title/Abstract])) OR (Acute coronary syndrome[Title/Abstract])) OR (Percutaneous coronary intervention[Title/Abstract])).

- 1. **Search strategy for Cochrane Library:**

#1 (Transfusion):ti,ab,kw OR (Blood transfusion):ti,ab,kw OR (Red blood cell transfusion):ti,ab,kw

#2 (Myocardial infarction):ti,ab,kw OR (Acute myocardial infarction):ti,ab,kw OR (ST-segment elevation myocardial infarction):ti,ab,kw OR (Non-ST-segment elevation myocardial infarction):ti,ab,kw OR (Acute coronary syndrome):ti,ab,kw

#3 (Percutaneous coronary intervention):ti,ab,kw

#4 #2 OR #3

#5 #1 AND #4

- 1. **Search strategy for EMBASE:**

#1 “Transfusion”:ab,ti

#2 “Blood transfusion”:ab,ti

#3 “Red blood cell transfusion”:ab,ti

#4 “Myocardial infarction”:ab,ti

#5 “Acute myocardial infarction”:ab,ti

#6 “ST-segment elevation myocardial infarction”:ab,ti

#7 “Non-ST-segment elevation myocardial infarction”:ab,ti

#8 “Acute coronary syndrome”:ab,ti

#9 “Percutaneous coronary intervention”:ab,ti

#10 #1 OR #2 OR #3

#11 #4 OR #5 OR #6 OR #7 OR #8 OR #9

#12 #10 AND #11

- 1. **Search strategy for Web of Science:**

#1 TI=(Transfusion OR Blood transfusion OR Red blood cell transfusion) OR AB= (Transfusion OR Blood transfusion OR Red blood cell transfusion)

#2 TI=(Myocardial infarction OR Acute myocardial infarction OR ST-segment elevation myocardial infarction OR Non-ST-segment elevation myocardial infarction OR Acute coronary syndrome OR Percutaneous coronary intervention) OR AB=(Myocardial infarction OR Acute myocardial infarction OR ST-segment elevation myocardial infarction OR Non-ST-segment elevation myocardial infarction OR Acute coronary syndrome OR Percutaneous coronary intervention)

#3 #1 AND #2

- 1. **Search strategy for Clinicaltrials.gov:**

Transfusion OR Blood transfusion OR Red blood cell transfusion | Myocardial infarction OR Acute myocardial infarction OR ST-segment elevation myocardial infarction OR Non-ST-segment elevation myocardial infarction OR Acute coronary syndrome OR Percutaneous coronary intervention

# Definition

**1.1 All-cause mortality:** All of the deaths that occur in the included population, regardless of the cause.

**1.2 Overall mortality:** It includes in-hospital all-cause mortality and follow-up all-cause mortality.

**1.3 In-hospital mortality:** All-cause mortality during hospitalization.

**1.4 Follow-up mortality:** All-cause mortality during the follow-up period.

**1.5 Follow-up reinfarction:** reinfarction during the follow-up period.

**1.6 Follow-up stroke:** stroke during the follow-up period.

**1.7 Follow-up heart failure:** heart failure during the follow-up period.

# Figure S1 Results of quality assessment using the Cochrane Collaboration’s risk of bias tool (Randomized Controlled Trial)

**
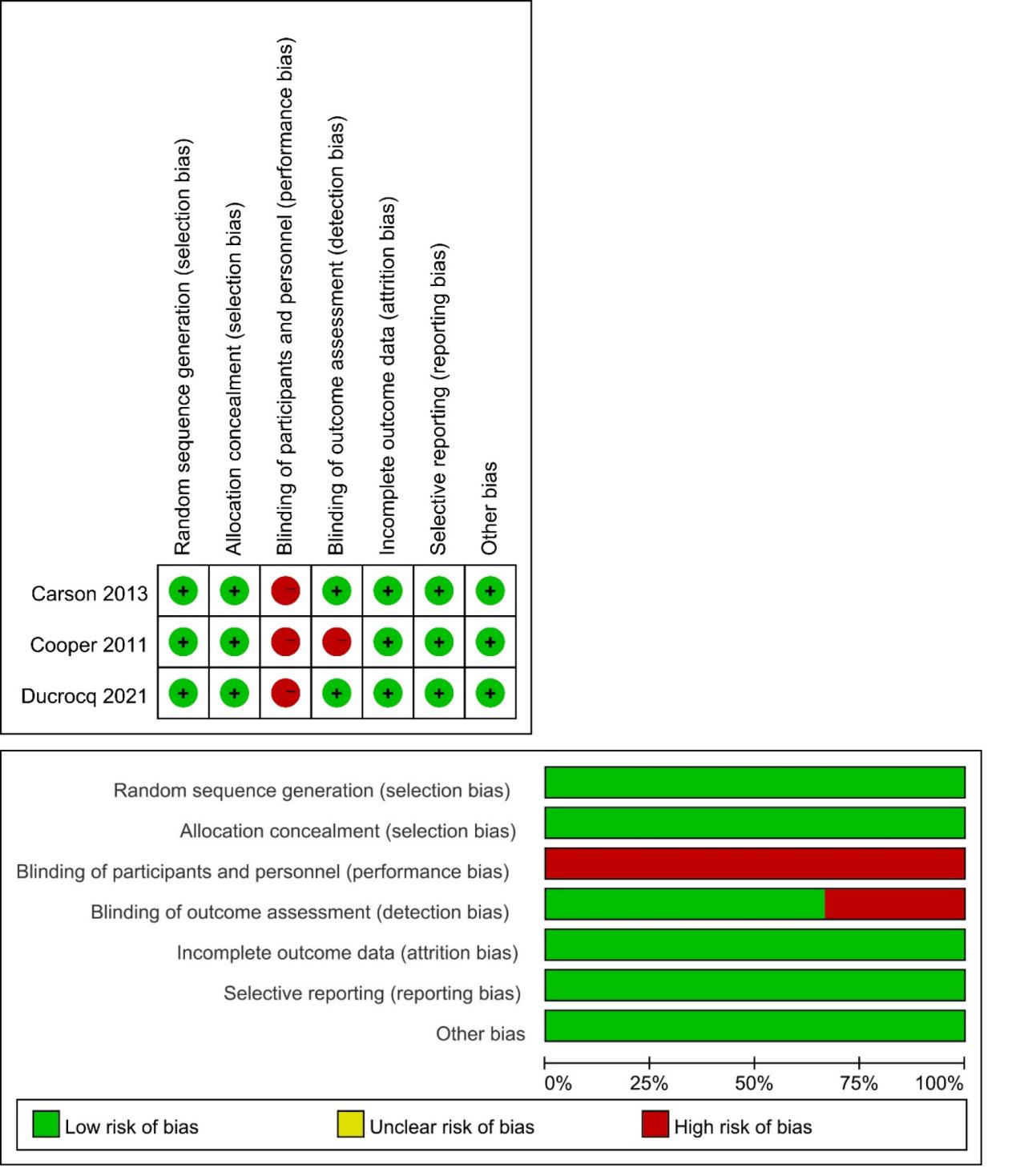
**

# Figure S2 Forest plot for the association between blood transfusion strategies with in-hospital and follow-up mortality


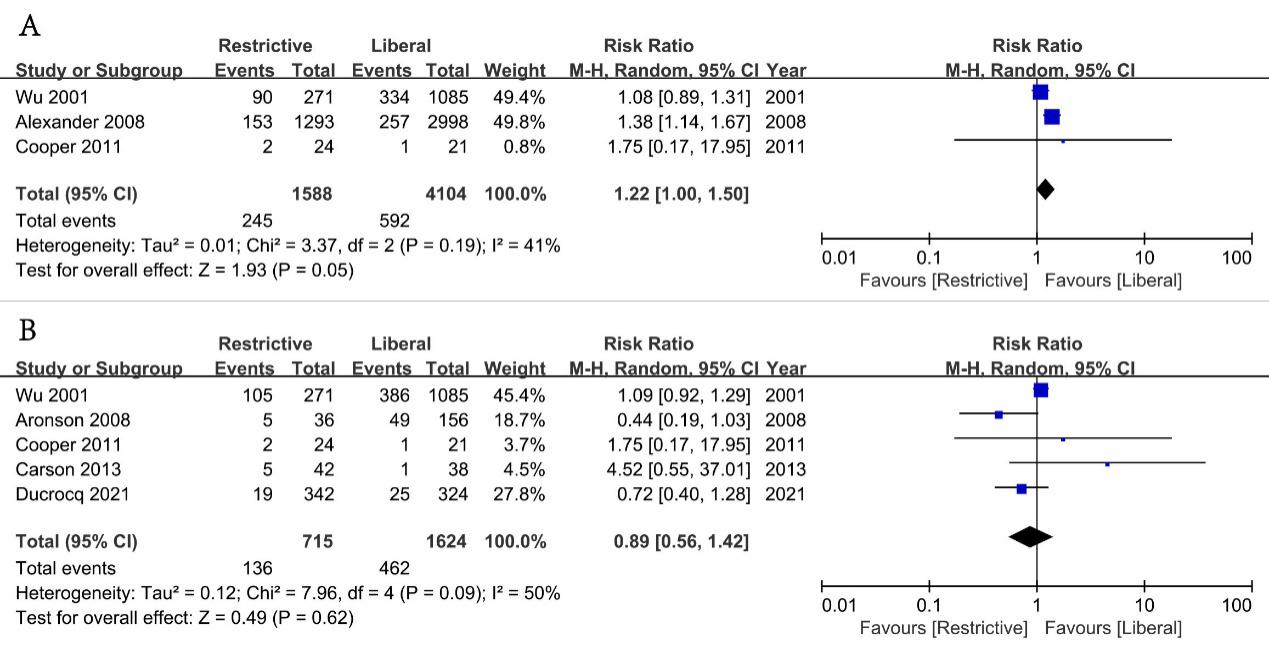


A: In- hospital mortality; B: Follow-up mortality

# Figure S3 Funnel plot analysis of potential publication bias


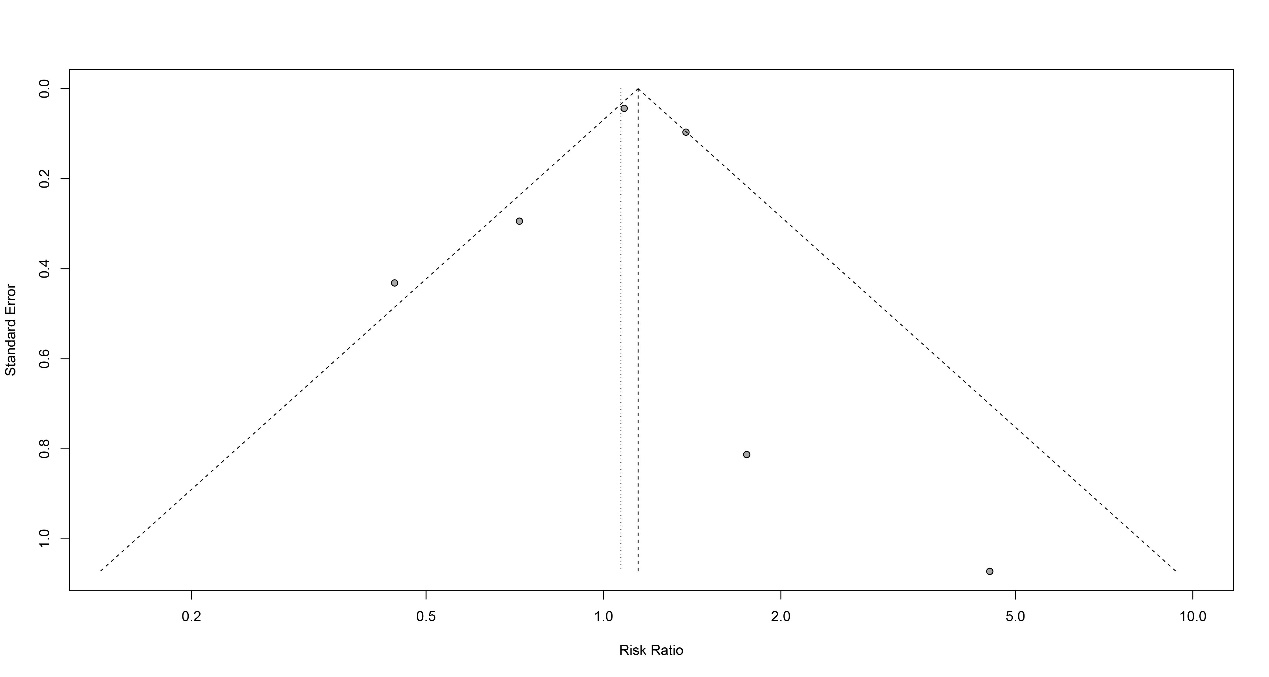


# Figure S4 A leave-one-out sensitivity analysis of this meta-analysis


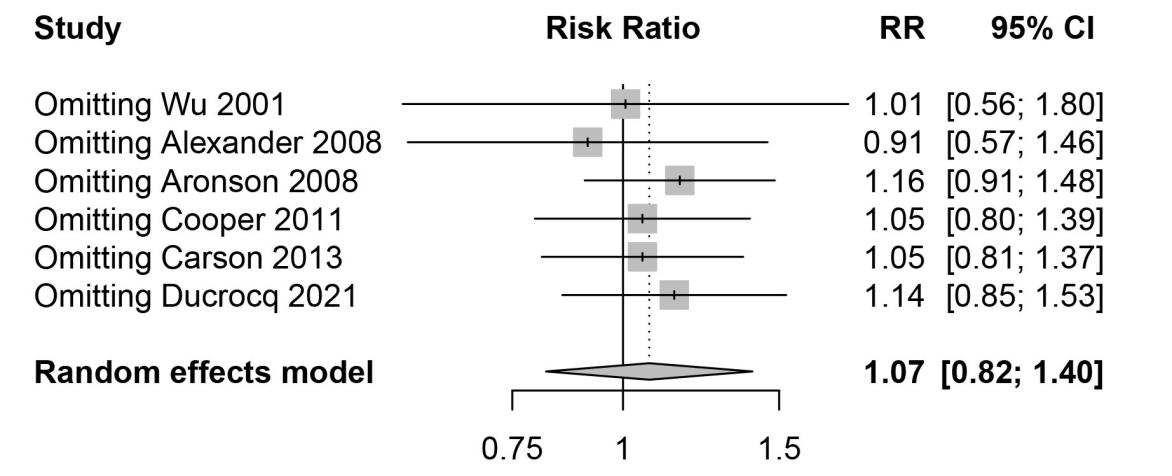

Supplement: Supplementary file 1 [file Data_Sheet_1.docx]
